# Supplementary material for: Epidermal injury-induced derepression of key regulator ATML1 in newly exposed cells elicits epidermis regeneration
Source: Nat Commun. 2023 Feb 23;14:1031. doi: 10.1038/s41467-023-36731-6 (PMC9950045; doi:10.1038/s41467-023-36731-6)
Supplement: Supplementary file 1 — Supplementary Information [file 41467_2023_36731_MOESM1_ESM.pdf]

Supplementary Fig. 1

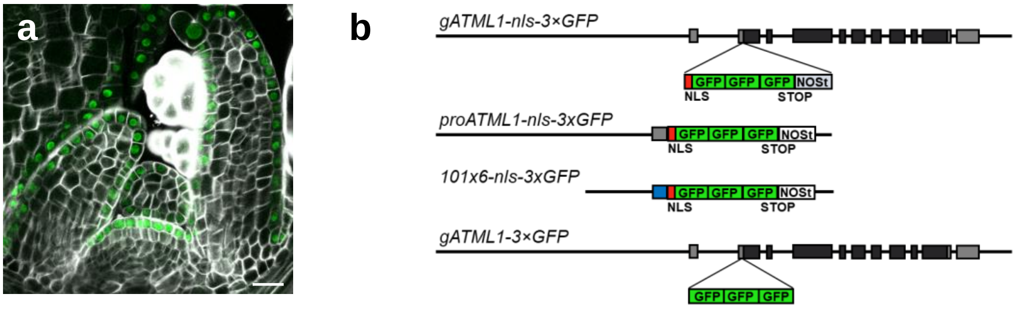

Supplementary Fig. 1 Visualization of *ATML1* activity.
a, A shoot apex of a 6-day-old *proATML1-nls-3xGFP* seedling. Green, GFP; white, SR2200. Scale bar: 20 µm. **b,** Constructs to visualize *ATML1* transcriptional activity and ATML1 protein localization. Triple *GFP* with a nuclear localization signal sequence is expressed under the whole *ATML1* regulatory sequence (top; *gATML1-nls-3xGFP*), the 3.4-kb sequence upstream of *ATML1* (second; *proATML1-nls-3xGFP*), and the 101-bp sequence (third; *101x6-nls-3xGFP*)[12,13]. The *ATML1* coding sequence translationally fused to the triple *GFP* gene is expressed under the whole *ATML1* regulatory sequence (bottom; *gATML1-3xGFP*)[12]. Gray boxes, untranslated regions; black boxes, coding regions; blue box, a cauliflower mosaic virus 35S minimal promoter. NLS, SV40 (large T-antigen) nuclear localization signal; GFP, green fluorescent protein; STOP, stop codon; NOSt, nopaline synthase terminator.

Supplementary Fig. 2

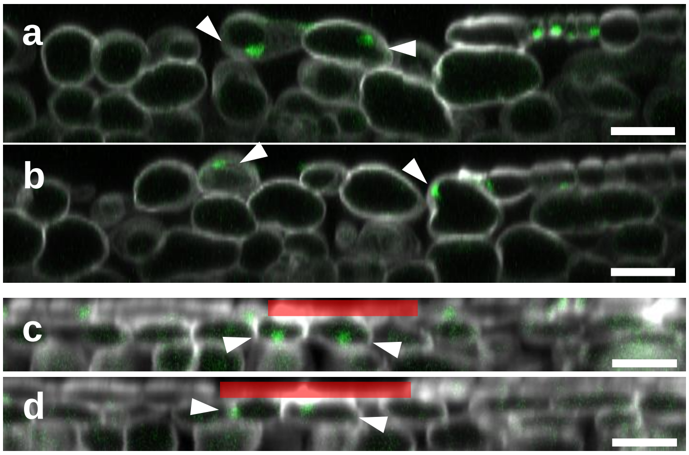

Supplementary Fig. 2 *ATML1* was induced only in the outermost mesophyll cells.

a–d, Optical cross sections of *proATML1-nls-3xGFP* leaves one day after removal of the epidermis (**a,b**) and one day after damaging the epidermis (**c,d**). After removal/damaging of the first or second leaf epidermis in 9-day-old seedlings, the seedlings were grown for 24 hours. Nine out of 10 peeled leaves showed the *ATML1* induction only in the outermost mesophyll cells. Green, GFP; white, SR2200; red lines, the damaged epidermis; white arrowheads, *ATML1*-positive mesophyll cells. Scale bars: 20 µm.

Supplementary Fig. 3

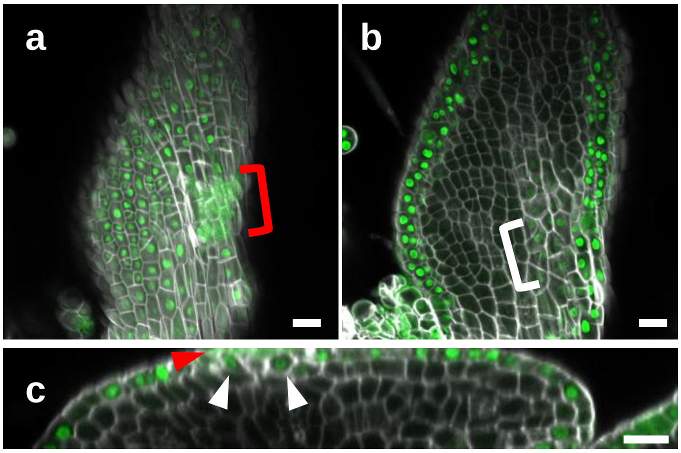

Supplementary Fig. 3 *ATML1* was induced in subepidermal cells beneath the damaged outer tissues in an immature leaf.

a,b, *proATML1-nls-3xGFP* signals in a damaged leaf. The first and second leaves in a 4-day-old seedling were damaged with a needle and grown for 24 hours. Confocal images of the *ATML1* reporter expression in the partly damaged epidermis (**a**) and in the underlying subepidermis (**b**), taken from the same leaf with different focal planes, are shown. **c**, Optical cross section of the leaf shown in **a,b**. Ten out of 12 leaves showed the *ATML1* induction in the subepidermal cells after damaging. Green, GFP or autofluorescence from damaged cells in **a**; white, SR2200; red bracket/arrowhead, the damaged epidermis; white bracket/arrowheads, *ATML1*-positive subepidermal cells. Scale bars: 20 µm.

Supplementary Fig. 4

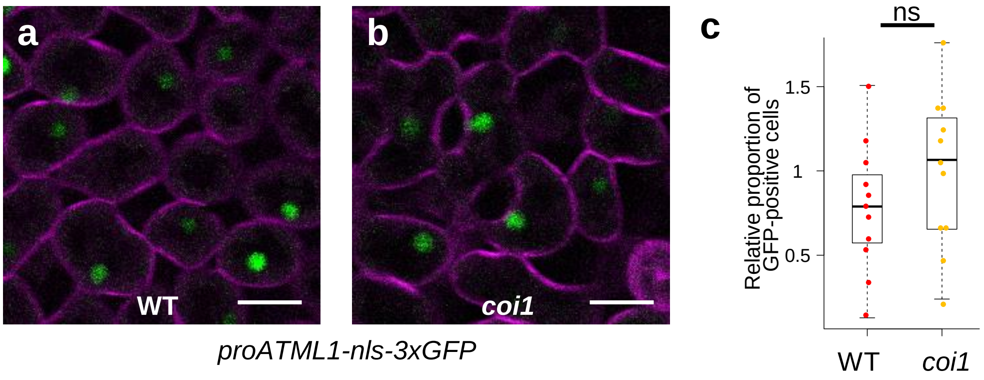

Supplementary Fig. 4 *ATML1* induction in mesophyll cells was not affected by the *coi1* mutation.

a,b, *proATML1-nls-3xGFP* signals were observed in the outermost mesophyll cells of 10-day-old seedlings of the wild type (WT; **a**) and *coi1* (**b**) at 1 dar. Experiments in **a**,**b** were repeated four times with similar results. **c,** The relative proportion of mesophyll cells showing GFP signals above the threshold was quantified in the wild type and *coi1* at 1 dar. n = 11 biologically independent leaves. Two-tailed Wilcoxon rank-sum test was used (ns, $P \geq 0.05$). In the box plots, the 25th percentile, the 50th percentile (central value) and the 75th percentile are marked by horizontal lines within the box. The ends of the whiskers indicate the maximum and minimum values within 1.5 x IQR from the box ends. Green, GFP; magenta, FM4-64. Scale bars: 20 μm.

Supplementary Fig. 5

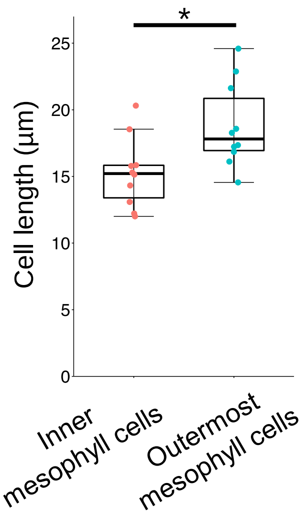

Supplementary Fig. 5 Cell length of the mesophyll cells exposed to the surface was longer than that of mesophyll cells beneath the epidermis.

Cell length of the L2 mesophyll cells exposed to the surface (outermost mesophyll) or overlaid by the epidermis (inner mesophyll) was measured in the dorsoventral direction in the first or second leaves of 10-day-old seedlings at 1 dar. n = ten biologically independent leaves; five and ten cells were measured in each leaf for inner mesophyll cells and outermost mesophyll cells, respectively. Two-tailed Welch's t-test was used (*, $P < 0.05$). In the box plots, the 25th percentile, the 50th percentile (central value) and the 75th percentile are marked by horizontal lines within the box. The ends of the whiskers indicate the maximum and minimum values within 1.5 x IQR from the box ends. An outlier is shown above the whisker. Each dot indicates average cell length in each leaf.

Supplementary Fig. 6

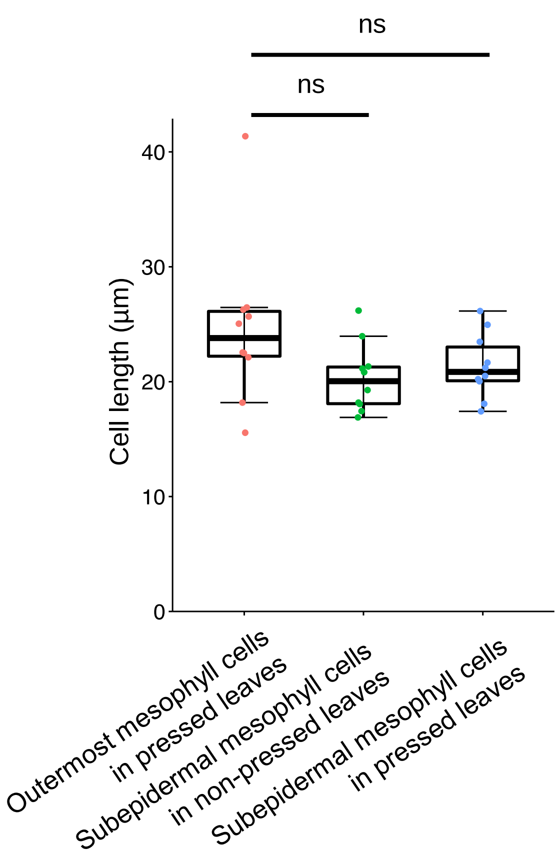

Supplementary Fig. 6 Mechanical pressure with the coverslips/paper clip suppressed the mesophyll cell elongation caused by the removal of the epidermis.

Cell length of the mesophyll cells in pressed/non-pressed leaves was measured in the dorsoventral direction in 10-day-old *proATML1-nls-3xGFP* seedlings at 1 dar. The first or second leaves were used. Each dot indicates average length of cells from a leaf. 'Subepidermal mesophyll cells' are the mesophyll cells that remain overlaid by the epidermis after the surgery. n = ten biologically independent leaves; five cells were examined in each leaf. A one-way analysis of variance (ANOVA) with the Tukey-Kramer test was used (ns, $P \geq 0.05$). In the box plots, the 25th percentile, the 50th percentile (central value) and the 75th percentile are marked by horizontal lines within the box. The ends of the whiskers indicate the maximum and minimum values within 1.5 x IQR from the box ends. Outliers are shown above and below the whiskers.

Supplementary Fig. 7

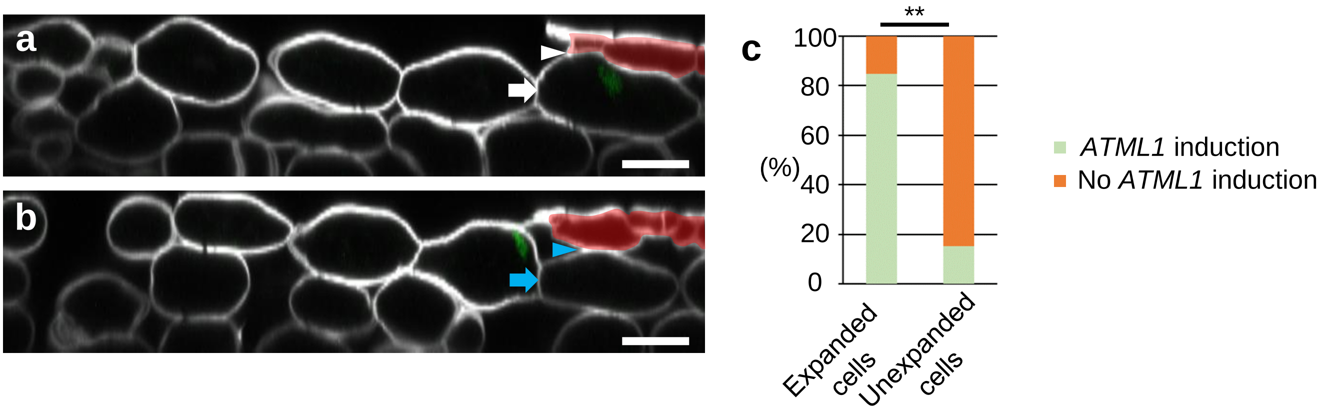

Supplementary Fig. 7 Mesophyll cell invasion into the epidermal layer was positively correlated with *ATML1* induction.

a,b, Optical transverse sections of the first or second leaf from a 10-day-old *proATML1-nls-3xGFP* seedling at 1 dar. Some “boundary mesophyll cells”, which are located near the boundary between peeled/unpeeled regions and are still overlaid with the epidermis, showed invasion into the epidermal layer. The boundary mesophyll cell invaded into the epidermal layer in (**a**) showed *ATML1* expression, whereas the uninvaded mesophyll cell in (**b**) did not. Red, epidermal cells that overlay the boundary mesophyll cells. White and blue arrows indicate invaded and uninvaded mesophyll cells, respectively. White and blue arrowheads indicate the outermost ends of invaded and uninvaded mesophyll cells, respectively. Note that the white arrowhead, but not the blue arrowhead, is located upper than the inner ends of the epidermal cells. Experiments in **a**,**b** were repeated twice with similar results. **c,** Frequency of *ATML1*-positive boundary mesophyll cells. Eleven out of 13 invaded cells and 10 out of 65 uninvaded cells showed *ATML1* induction (green) at 1 dar. Fisher’s exact test was used (**, $P < 0.01$). Green, GFP; white, SR2200. Scale bars: 20 μm.

Supplementary Fig. 8

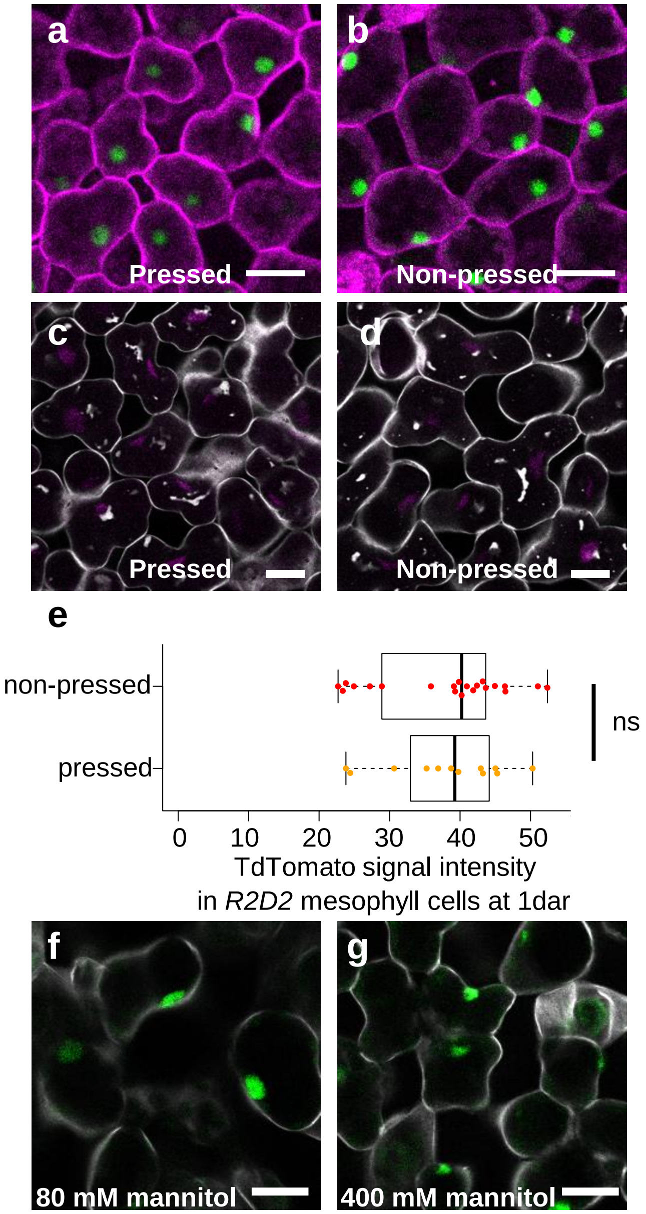

Supplementary Fig. 8

Supplementary Fig. 8 The cell viability of mesophyll cells was not impaired by the pressure or mannitol treatment.

a,b, *GFP-ATML1* signals in the outermost mesophyll cells with (**a**) or without (**b**) pressure treatment at 1 dar. A 9-day-old *RPS5A>>GFP-ATML1* seedling, with a peeled leaf pressed between coverslips, was grown in liquid MS medium supplemented with 10 µM estradiol for 24 hours starting from the time of epidermis removal. *GFP-ATML1* induction was not inhibited by the pressure treatment. **c,d,** *RPS5A-mDII-nls-TdTomato* signals in the outermost mesophyll cells of pressed (**c**) and non-pressed (**d**) *R2D2* leaves at 1 dar. After the first or second leaf epidermis in 9-day-old seedlings was peeled, the seedlings were grown with or without pressure treatment for 24 hours. **e,** *RPS5A-mDII-nls-TdTomato* signals at 1 dar quantified in the outermost mesophyll cells in non-pressed and pressed leaves of *R2D2* seedlings grown as indicated above. n = 21 biologically independent leaves for 'non-pressed' and n = 12 biologically independent leaves for 'pressed'. Two-tailed Welch's t-test was used (ns, $P \geq 0.05$). In the box plots, the 25th percentile, the 50th percentile (central value) and the 75th percentile are marked by horizontal lines within the box. The ends of the whiskers indicate the maximum and minimum values within 1.5 x IQR from the box ends. Each dot indicates an average signal intensity in a single leaf. **f,g,** After the epidermis of the first and second leaves was removed in 9-day-old *RPS5A>>GFP-ATML1* seedlings, the seedlings were grown in liquid MS medium supplemented with 10 µM estradiol and 80 mM (**f**) or 400 mM (**g**) mannitol for 24 hours. Nineteen out of 19 leaves or 22 out of 22 leaves showed *GFP-ATML1* induction in the outermost mesophyll cells of the seedlings grown in 80 mM or 400 mM mannitol-containing MS medium, respectively. Each experiment was repeated twice for **a**,**b** and three times for **c**,**d**,**f**,**g** with similar results. Green, GFP; magenta, FM4-64 in **a,b** and TdTomato in **c,d**; white, SR2200. Scale bars: 20 µm.

Supplementary Fig. 9

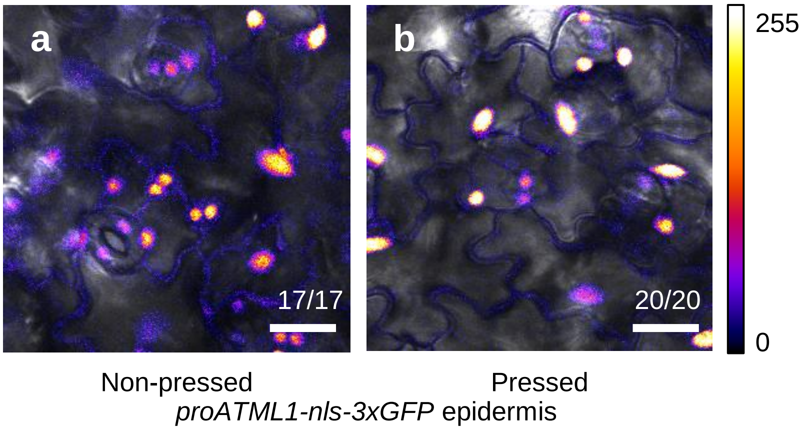

Supplementary Fig. 9 The pressure treatment did not inhibit *ATML1* expression in the differentiated epidermis.

a,b, *ATML1* promoter activity in the epidermis of the first or second leaves without (**a**) or with (**b**) pressure treatment. Nine-day-old seedlings were grown for 24 hours with or without their leaves pressed with coverslips and a paperclip. GFP signal intensities are shown according to the color map on the right. Transmitted light images (gray scale) were overlaid to show cell boundaries. Fractions on the panels indicate the proportion of leaves showing the similar expression as in the images. Experiments in **a**,**b** were repeated three times with similar results. Scale bars: 20 µm.

Supplementary Fig. 10

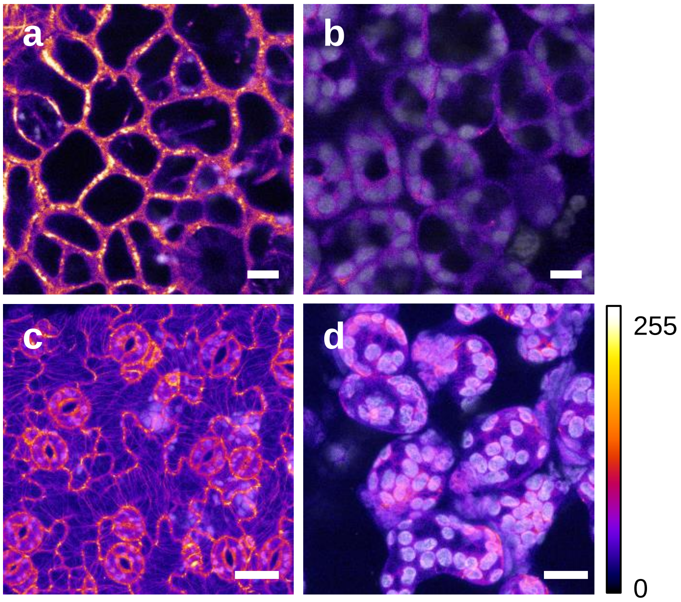

Supplementary Fig. 10 GFP-TUA6 localization in the leaves with and without removal of the epidermis.

a,b, GFP-TUA6 signals in the anticlinal cell boundaries of the epidermis (**a**) and the subepidermal mesophyll cells (**b**) in the first or second leaves of 6-day-old seedlings without surgery treatment. The subepidermal mesophyll cells in 16 out of 17 leaves did not show clear foci of GFP-TUA6 signals. **c,d,** Maximum projection images of GFP-TUA6 signals in the epidermis (**c**) and the outermost mesophyll cells (**d**). GFP signals in the first or second leaves of 10-day-old seedlings without removal of the epidermis (**c**) or at 1 dar (**d**) are shown. The outermost mesophyll cells in 21 out of 26 leaves showed microtubule arrays one day after removal of the epidermis. Experiments in **a-d** were repeated three times with similar results. GFP signals were shown according to the color code on the right. Chlorophyll autofluorescence is shown in gray scale. Scale bars: 5 µm in **a,b** and 20 µm in **c,d**.

Supplementary Fig. 11

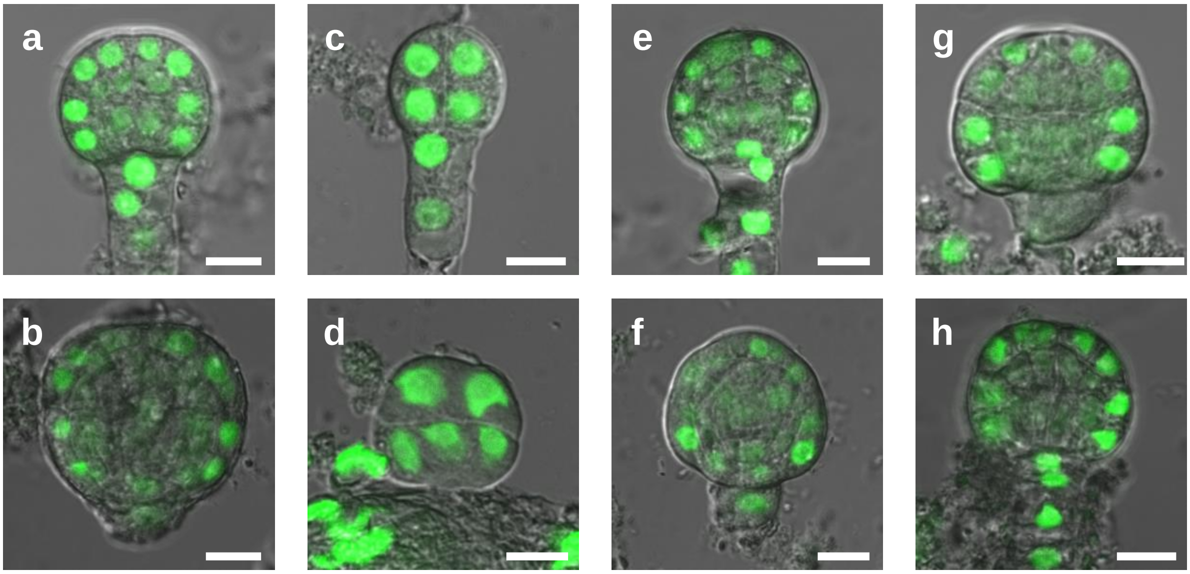

Mock Oryzalin 23/23 PD98050 16/16 MG132 14/16

proATML1-nls-3xGFP

Supplementary Fig. 11 The inhibitors that decrease de-novo *ATML1* induction in the mesophyll cells did not affect *ATML1* expression in embryos.
a–h, *proATML1-nls-3xGFP* signals in embryos. Ovules of *proATML1-nls-3xGFP* plants were incubated for 24 hours in liquid medium supplemented with 0.1% DMSO (**a**,**b**, Mock), 30 µM oryzalin (**c**,**d**), 25 µM PD98059 (**e**,**f**) or 10 µM MG132 (**g**,**h**). Experiments in **a-h** were repeated twice with similar results. Green, GFP. Fractions on the panels indicate the proportion of embryos displaying the same expression pattern as shown in the images. Scale bars: 10 µm.

Supplementary Fig. 12

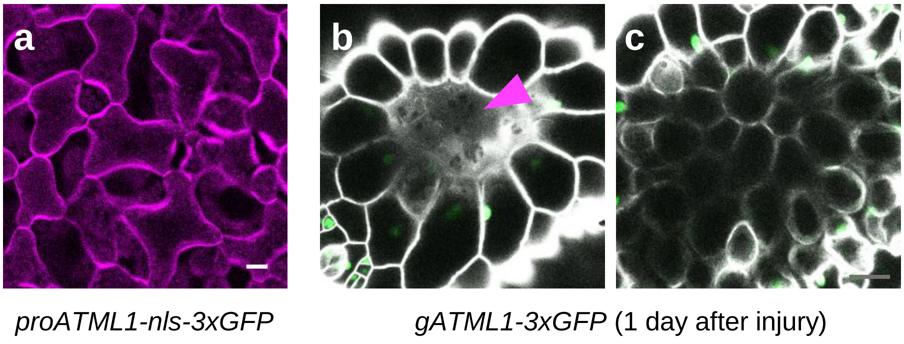

Supplementary Fig. 12 Mesophyll cells of 9-day-old seedlings did not regenerate the epidermis.

a, Mesophyll cells observed seven days after removal of the epidermis. After the first or second leaf epidermis of 9-day-old *proATML1-nls-3xGFP* seedlings was removed, the seedling was grown on the MS-phytagel plate for seven days. **b,c,** *gATML1-3xGFP* signals in a leaf after trichome injury. After a trichome of the first or second leaf in a 9-day-old *gATML1-3xGFP* seedling was killed with a needle, the seedling was grown on an MS-phytagel plate for 24 hours. Epidermal cells (**b**) with the damaged trichome (arrowhead) and mesophyll cells (**c**) beneath **b** are shown. Each experiment was repeated three times for **a** and twice for **b**,**c** with similar results. Green, GFP in **b,c**; magenta, FM4-64 in **a**; white, PI in **b,c**. Scale bars: 20 µm.

Supplementary Fig. 13

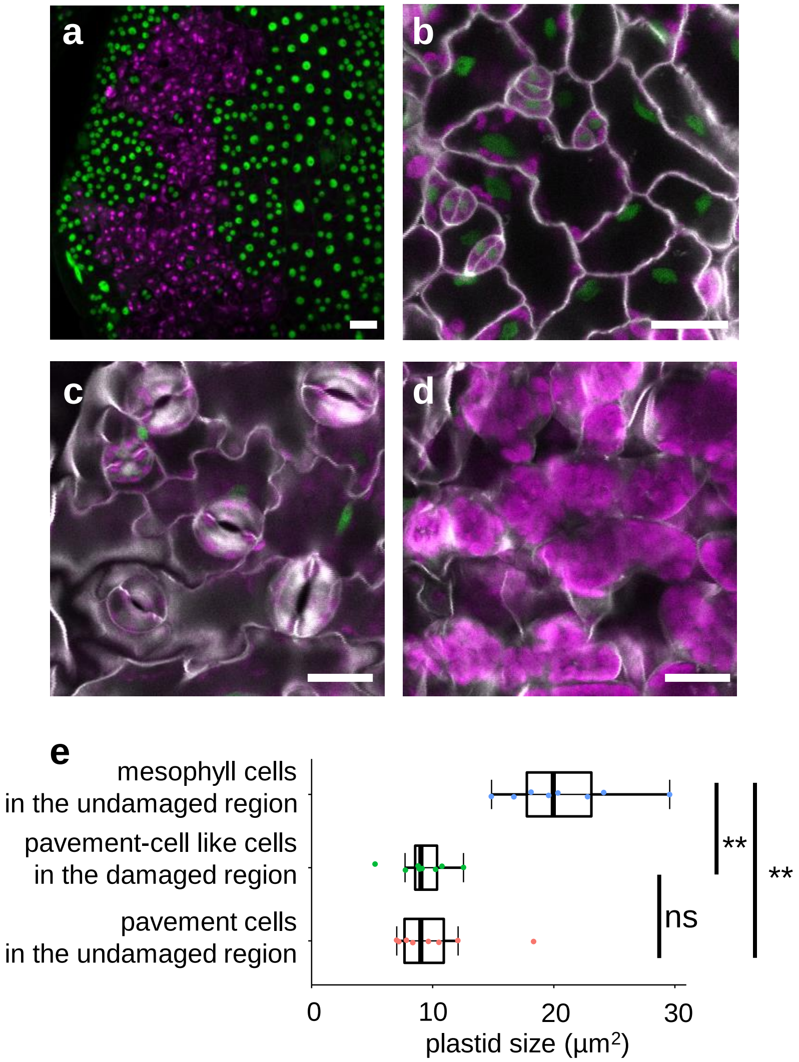

Supplementary Fig. 13 The epidermis was regenerated in the outermost position after the surgical injury.

a,b, Damaged *proATML1-nls-3xGFP* leaves. Right after the injury of abaxial outer tissues, 6-day-old seedlings were stained with PI to visualize dead cells (**a**). Five days after the wounding, pavement cell-like cells showing *proATML1-nls-3xGFP* expression and small lobes appeared in the damaged region (**b**). **c,d,** Plastid autofluorescence in the undamaged region of *proATML1-nls-3xGFP* leaves five days after the injury. Epidermal cells (**c**) and mesophyll cells (**d**) beneath **c** are shown. Experiments in **a**-**d** were repeated three times with similar results. **e,** Plastid sizes in the outermost cells of the undamaged/damaged regions and in mesophyll cells of undamaged regions. n = eight biologically independent leaves (three or four plastids were measured and averaged in each cell; two or three cells were examined in each leaf). One-way ANOVA with the Tukey-Kramer test was used (ns, $P \geq 0.05$; **, $P < 0.01$). In the box plots, the 25th percentile, the 50th percentile (central value) and the 75th percentile are marked by horizontal lines within the box. The ends of the whiskers indicate the maximum and minimum values within 1.5 x IQR from the box ends. Outliers are shown above and below the whiskers. Each dot indicates an average plastid size in a single leaf. Green, GFP; white, SR2200; magenta, PI in **a** and plastid autofluorescence in **b**–**d**. Scale bars: 20 μm.

Supplementary Fig. 14

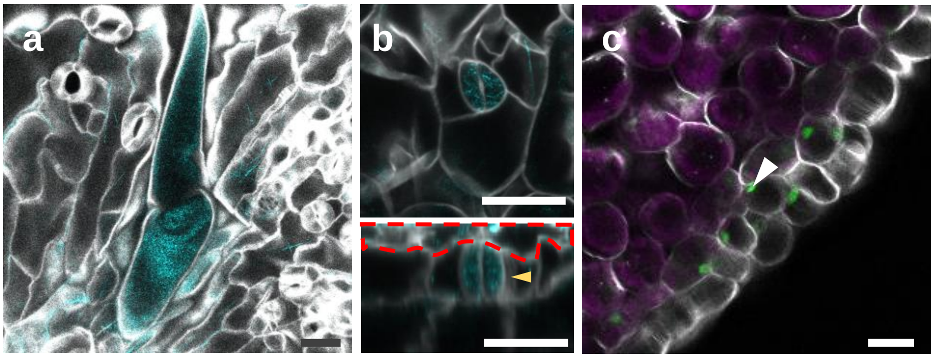

Supplementary Fig. 14 Guard cell, trichome and pavement cell identities were detected in the damaged leaves.

a, *proGL2-GUS* positive trichome-like cell formed in the damaged region. Note that this trichome-like cell is less branched and not surrounded by typical accessory cells. Fourteen out of 25 damaged leaves formed the similar trichome-like cells as shown in the image. **b,** *KAT1*-positive guard cells in the inner tissue viewed from the surface of the leaf (upper). Optical cross section of an injured *proKAT1-GUS* leaf (lower). The red dotted line delineates the damaged epidermal layer. The yellow arrowhead indicates *KAT1*-positive guard cells in the inner tissue of the leaf. Eleven out of 79 damaged leaves showed guard cell formation in the inner tissue. **c,** Histological section of a *proATML1-nls-3xGFP* leaf. The white arrowhead indicates an *ATML1*-positive cell that is overlaid with the epidermis and shows less chlorophyll autofluorescence. Among 216 *ATML1*-positive inner cells, 36 cells showed less chlorophyll autofluorescence compared with the intact mesophyll cells. After the adaxial outer tissue of the first and second leaves was damaged in 6-day-old seedlings, the seedlings were grown for five days (**a**–**c**). Each experiment was repeated three times for **a**,**c** and four times for **b** with similar results. Blue, GUS; green, GFP; white, SR2200; magenta, chlorophyll autofluorescence. Scale bars: 20 µm.

Supplementary Fig. 15

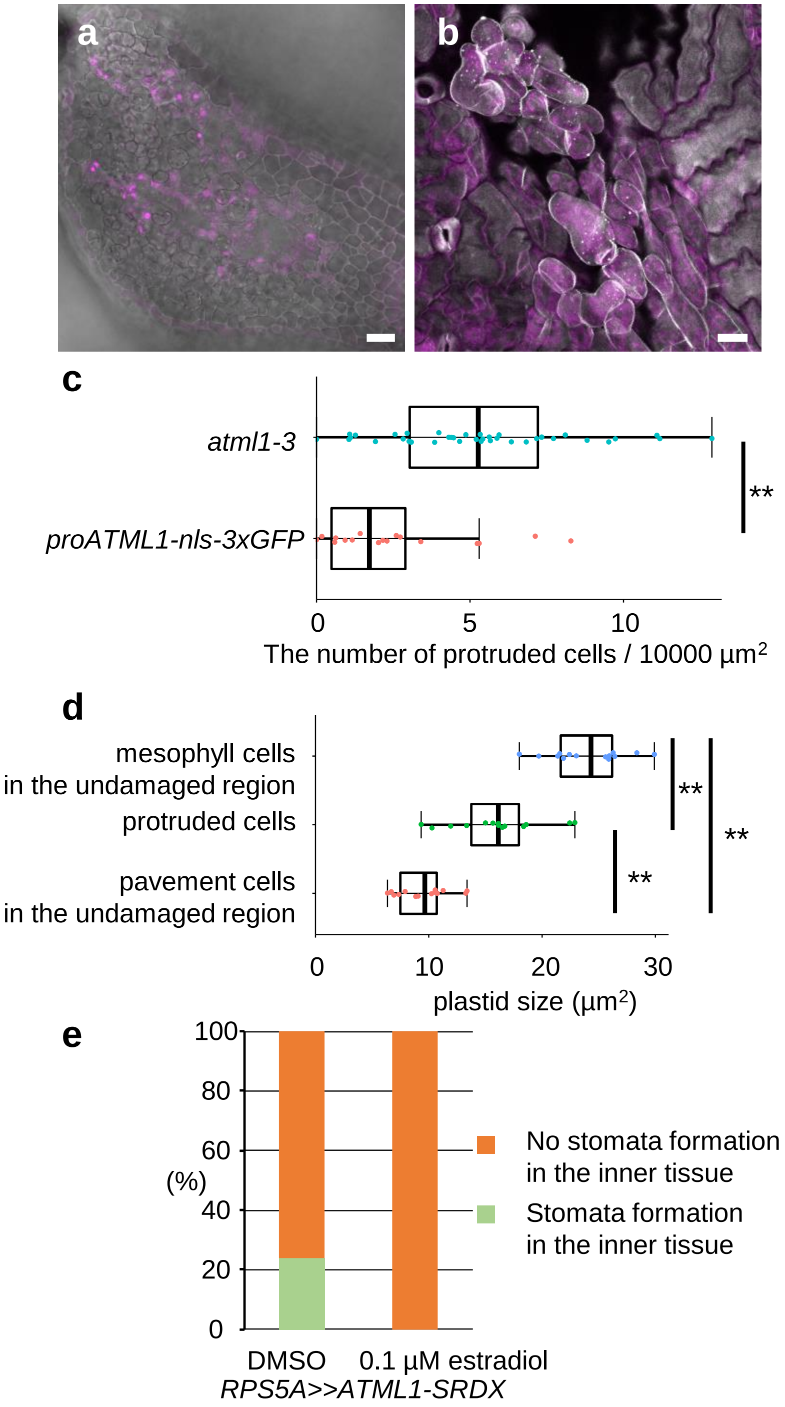

Supplementary Fig. 15

Supplementary Fig. 15 *ATML1* and/or its downstream genes were required for the epidermis regeneration after the surgical injury.

a,b, Damaged *atml1-3* leaves. Right after the injury of abaxial outer tissues of the first and second leaves in 6-day-old seedlings, the damaged seedlings were stained with PI. The transmitted light image (gray scale) was overlaid with PI signals (**a**). Five days after the wounding, cells in the damaged region protruded from the surface and were distinct from the epidermis in terms of morphology (**b**). **c,** The density of protruded cells in the damaged regions of *proATML1-nls-3xGFP* and *atml1-3* leaves. n = 20 biologically independent leaves for *proATML1-nls-3xGFP* and n = 41 biologically independent leaves for *atml1-3*. Two-tailed Wilcoxon rank-sum test was used (**, $P < 0.01$). Each dot indicates the density of protruded cells in a single leaf. **d,** Plastid sizes in the pavement cells, protruded cells and mesophyll cells of *atml1-3* leaves. n = 16 biologically independent leaves for pavement and protruded cells and n = 14 biologically independent leaves for mesophyll cells. The size of three or four plastids was measured and averaged in each cell. Three cells were examined in each leaf. One-way ANOVA with the Tukey-Kramer test was used (**, $P < 0.01$). Each dot indicates an average plastid size in a single leaf. For all the box plots, the 25th percentile, the 50th percentile (central value) and the 75th percentile are marked by horizontal lines within the box. The ends of the whiskers indicate the maximum and minimum values within 1.5 x IQR from the box ends. Outliers are shown above the whisker. **e,** Frequency of guard cell formation in the inner tissue of the damaged *RPS5A>>ATML1-SRDX* leaves (green). The abaxial outer tissues of the first and second leaves in 6-day-old *RPS5A>>ATML1-SRDX* seedlings were damaged with a needle. After damaging, the seedlings were moved to the MS plate supplemented with DMSO or 0.1 μM estradiol and grown for five days. Eleven out of 46 leaves showed inner guard cell formation in DMSO-treated seedlings whereas none of 32 leaves showed inner guard cell formation in estradiol-treated seedlings. n = 46 biologically independent leaves for DMSO and n = 32 biologically independent leaves for estradiol. Magenta, PI in **a** and plastid autofluorescence in **b**; gray, SR2200. Scale bars: 20 μm.
